# Store-and-forward teledermatology: a case report

**DOI:** 10.1186/1756-0500-7-588

**Published:** 2014-09-01

**Authors:** Matthew A Lenardis, Robert S Solomon, Fok-Han E Leung

**Affiliations:** 1 King’s College Circle, M5S 1A8 Toronto, ON Canada; 80 Bond Street, M5B 1X2 Toronto, ON Canada

**Keywords:** Teledermatology, Telemedicine, Family medicine, Dermatology, Homeless

## Abstract

**Background:**

Telemedicine is increasingly being used as part of routine practice for many physicians and healthcare providers across the country. Due to its visual nature, dermatology is ideally suited to benefit from this new technology. The use of teledermatology (telemedicine in dermatology) in a primary care setting allows for an expert opinion without the need for an in-person referral. Furthermore, it can improve patient access in remote areas. Store-and-forward teledermatology is the most commonly employed method.

**Case presentation:**

This case describes a Caucasian male in his fifties with no fixed address or telephone number who presented to his family doctor with an enlarging nevus on his chest, and required a dermatology referral. Given these limitations, a traditional fax and phone referral would not be possible. Instead store-and-forward teledermatology was employed. It was then determined by the dermatologist that the nevus was benign and did not require treatment.

**Conclusion:**

This case demonstrates the utility of store-and-forward teledermatology in what is unfortunately not an uncommon scenario in Canada. The patient was successfully managed, and a logistically difficult and expensive in-person referral was avoided.

## Background

Recent advances in technology have allowed for a broadened use of telehealth across the country. From psychiatry to anesthesia [1, 2], telemedicine is increasingly being used as part of routine practice for many physicians and healthcare providers. Due to its visual nature, dermatology is an ideal candidate for the use of telemedicine. The use of teledermatology (telemedicine in dermatology) in a primary care setting allows for an expert opinion without the need for a referral. Furthermore, it can improve patient access in remote areas. There currently exist two major forms of teledermatology: store-and-forward (S&F) and live interactive (LI).

S&F teledermatology conventionally requires the primary care physician to take photographs of the lesion or affected area, and then digitally transfer them to the dermatologist by email or secure servers. The dermatologist then responds with the diagnosis and recommendations. S&F teledermatology has been shown to have comparable diagnostic accuracy to conventional methods while improving patient access, being cost effective, and even improving remote medical education [3–5]. Furthermore, S&F teledermatology has been shown to not have significant differences in clinical outcomes as compared to traditional referrals [6, 7].

In contrast, LI teledermatology involves a live videoconference between the referring physician and patient on one end, and dermatologist on the other. LI teledermatology enables real-time discussion amongst all parties and facilitates education of both patient and physician [8]. It also allows for instant clarification of any issues that may arise. However, unlike S&F teledermatology, LI methods require the coordination of both primary care physician and dermatologist, and rely much more significantly on proper functioning of technology and security. S&F teledermatology is therefore much more commonly employed.

## Case presentation

A Caucasian male patient in his fifties presented to his family doctor for a routine physical examination. He had been a patient of the family practice for several years, and his preventative care was up to date. His only medical condition was a seizure disorder, and this was well controlled with medication.

On physical exam, his vital signs were stable and his heart and lungs both sounded normal on auscultation. His abdomen was soft and non-tender. When doing a skin check, a 6x6mm nevus was observed on his chest. It was slightly raised and there was a dark, irregular pigmentation throughout. There was no pain or itching associated with the lesion. While the patient reported that it had been present since childhood, it had recently grown and become more pigmented. The borders of the lesion were well defined.

A dermatological referral was considered. However, the patient was living without a fixed address, and was staying in various shelters and occasionally sleeping on friends’ couches. He also did not have access to a phone or a phone number. The appointment booking staff indicated that a referral to dermatology in this case would not be possible given these limitations.

### Solution

Instead of trying to arrange an in-person appointment with a dermatologist which would require faxing, phoning or mailing correspondence to the patient, S&F teledermatology was employed. Images were taken of the nevus and sent to a dermatologist for consultation. The physician took the photos and wrote a brief electronic note. The clinic administrative assistant then inputted all the information into the provincial telemedicine portal, which was then uploaded to the dermatologist’s office. When the dermatologist replied with his diagnosis, it was determined that the nevus was benign and did not require treatment.

## Discussion

This case highlights one of the unique advantages of teledermatology and telemedicine in general. It is estimated that at least 200,000 Canadians experience homelessness each year, and as many as 30,000 people will be homeless on any given night [9]. Given this high figure, it falls on the physician to seek treatment options that are suitable to the patient context. In a situation where a referral is required, the matter is greatly complicated when the patient has no fixed address, and therefore cannot be contacted for the referral or follow-up. As illustrated in the case, similar to remote and rural populations, inner city populations are particularly vulnerable. S&F teledermatology was suitable in this case because it accommodated the patient’s lack of accessibility while still maintaining the standard of care. A previous study found that more than 75% of patients that were cared for through teledermatology were at or below the federal poverty level, and were often located in rural and isolated areas that would otherwise limit their access to a dermatologist [10].

S&F teledermatology is being increasingly implemented in centers across the country, but there remain some barriers that limit its usage. Cost is often an issue whenever new technologies emerge. Indeed, there appears to be conflicting evidence regarding the cost-efficiency of teledermatology [11–13]. It is important to note that the cost-efficiency varies greatly with the population that it is used in, so it is difficult to make a broad estimate.

In a case of no fixed address, travel is also of concern. Telemedicine has the ability to greatly reduce the need for patients to travel to seek care, especially in those that live in rural or very underserved areas. It has been shown that the use of S&F teledermatology is associated with 43% rate of avoided travel as compared to traditional methods. This rose to as much as 70% in LI cases [14]. For low-income or homeless patients, this avoidance of travel can also be of great importance due to the costs associated with simply getting to the referred physician.

## Conclusions

The patient in this case was successfully managed using S&F teledermatology. A logistically difficult (lack of a fixed address or telephone number) and expensive in-person referral was successfully avoided. More timely and equitable care was achieved through telemedicine.

## Consent

Written informed consent was obtained from the patient for publication of this case report. A copy of the written consent is available for review by the Editor of this journal.

## Authors’ information

MAL is a medical student at the University of Toronto in Toronto, Ontario. RSS is a consultant dermatologist at St. Michael’s Hospital and guest lecturer at the University of Toronto in Toronto, Ontario. FHL is a staff physician at the Health Centre at 80 Bond of St Michael’s Hospital in Toronto, Ontario.

## Abbreviations

S&F: Store and forward teledermatology
LI: Live-interactive teledermatology.
